# Trench Conflict with Combatants and Infectious Disease

**DOI:** 10.3201/eid2411.AC2411

**Published:** 2018-11

**Authors:** Terence Chorba

**Affiliations:** Centers for Disease Control and Prevention, Atlanta, Georgia, USA

**Keywords:** art science connection, emerging infectious diseases, Ernst Liebenauer, In Entrenchment, World War I, Trench Conflict with Combatants and Infectious Disease, World War I, influenza, influenza viruses, viruses, Bartonella, bacteria, trench conflict, combatants, about the cover

**Figure:**
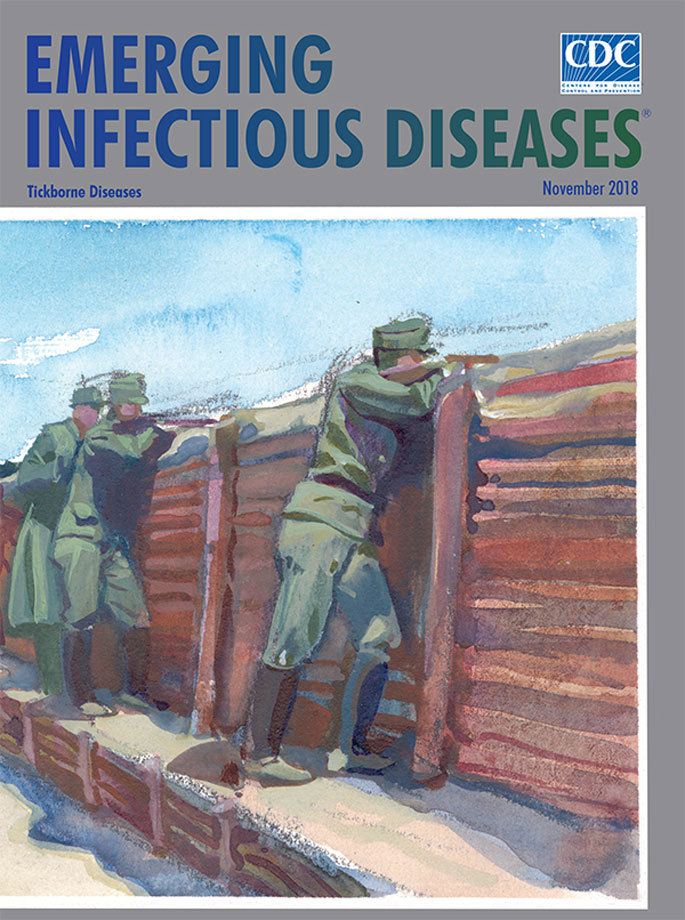
**Ernst Liebenauer (1884–1970), *In Entrenchment, World War I* (c. 1915).** Watercolor, pencil, and gouache on paper, 5.6 in x 4 in/14.2 cm x 10.1 cm. Digital image from private collection, Atlanta, Georgia, USA.

A century ago, the world was ensnared in the Great War, 1914–1918, now known as the First World War. During that war, an estimated 9 million combatants and as many as 7 million civilians died, and it brought to an end the German, Russian, Austro-Hungarian, and Ottoman Empires. Infectious diseases played a prominent role in that war, resulting in more casualties than did war-inflicted wounds. With several decades of knowledge about bacterial organisms, armies had implemented sanitation measures such as latrines and water purification methods to control diarrheal and dysenteric diseases. Vaccine successes had been documented for smallpox and typhoid. However, louse-borne typhus killed 2–3 million soldiers and civilians on the Eastern Front, and the war’s end in November 1918 was hastened by an influenza pandemic that had begun in January 1918 and eventually claimed the lives of an estimated 50 million.

Because of the huge numbers of casualties, control of media was important for maintaining positive public opinion and support for the war efforts; all combatant countries developed central censorship and propaganda offices. The United States entered the war in April 1917, fully two-and-a-half years into the conflict, and created its own Committee on Public Information and its own Censorship Board. For the Austro-Hungarian forces, the central censorship and propaganda institution was the War Press Office, or *Kriegspressequartier*, which eventually included more than 750 writers, journalists, photographers, and filmmakers, and some 150 visual artists. Painters and photographers worked in the field of combat, many as military officers, and sketched quick impressions, which they could later render more elaborate or refined, when they were away from the dangers of the front. Among these many painters was a young artist, Ernst Liebenauer (1884–1970), who had studied under reknowned realist Christian Griepenkerl at the *Wiener Akademie der Bildenden Künste* (Viennese Academy of Fine Arts) and later at the *Spezialschule für Historienmalerei* (Special School for Historical Painting) under another well-known Austrian portrait and landscape painter, Franz Rumpler. During the war, Liebenauer focused on military subjects, but after the war, he became a painter of landscapes, still life, portraits, and mythical scenes. He was best known as an illustrator of children’s books and fairy tales, including versions of Daniel Defoe’s *Robinson Crusoe* and the works of the Brothers Grimm.

On this month’s cover, Liebenauer’s impressionist watercolor sketch, *In Entrenchment, World War I*, presents an almost pristine, impressionist picture of the Austro-German trenches. The 5-year conflict was fought at sea and on land, but the principal battlefields emerged where opposing sides constructed elaborate lines of fortified trenches that meandered for two thousand miles on the Western Front, from the North Sea to the border of Switzerland with France. Between the opposing trenches was a “no man’s land” of barbed wire and mines. Liebenauer’s sketch portrays no carnage, no deprivation, no disorder. A long-coated military officer calmly supervises four soldiers, clad in orderly uniforms under a bright and colorful sky, as they fire rifles across no man’s land. What may be random, penciled sketch lines may also be intended to resemble the pervasive barbed wire of the trenches, and the blue, shadowed trough in parallel with the step up to the trench wall may be intended to convey an image of pooled water or snow. The overall mood is relatively tranquil. The sketch is inspirational, consistent with the desires of the *Kriegspressequartier*, featuring the bravery of the soldiers at the front, and devoid of the horrors of the conflict or the ubiquitous filth of the trenches.

There are conflicting theories as to the site of origin of the H1N1 strain of the influenza virus that swept across the world, infecting perhaps 500 million; but the spread of the epidemic was amplified by forced congregate settings, mass migrations, intercontinental traffic, and abject living conditions. Spain did not enter the conflict and was less engaged in requiring censorship or promoting propaganda. Consequently, the 1918 pandemic became known as “Spanish flu,” as its burden was reported more quickly and more extensively in the Spanish press.

In addition to trench warfare itself, World War I gave us trench-warfare disease terms: trench foot (or immersion foot, a noninfectious, nonfreezing, damp exposure injury that often led to gangrene, often necessitating amputations), and trench mouth (acute necrotizing ulcerative gingivitis, a painful, fast-moving, noncontagious infection by mostly anaerobic bacteria, particularly *Fusobacterium, Prevotella intermedia,* and spirochete species). It also gave us the term trench fever*,* the sudden onset of undulating fever, headache, and dizziness, caused by *Bartonella quintana* infection, for which the principal vector is the human body louse. Infection with *B. quintana* also causes endocarditis, chronic bacteremia, bacillary angiomatosis, and anomalous development of blood-filled cavities in the liver (peliosis hepatis).

This month’s centenary of the end of Great War reminds us of the wealth of antimicrobials and vaccines that the last century has brought. Unfortunately, with increasing antimicrobial drug resistance, we are also burdened with fear of a return to a setting in which we have few defenses against the most common of infectious disease foes.

## References

[1] Aichelburg W. Das Kriegspressequartier – KPQ (The War Office) [cited 2018 Sep 7]. http://www.wladimir-aichelburg.at/kuenstlerhaus/mitglieder/kriegspressequartier/

[2] Anstead GM. The centenary of the discovery of trench fever, an emerging infectious disease of World War 1. Lancet Infect Dis. 2016;16:e164–72. 10.1016/S1473-3099(16)30003-2 27375211 PMC7106389

[3] Cornwall M. The undermining of Austria-Hungary: the battle for hearts and minds. London: St. Martin’s Press; 2000. p. 16–39.

[4] Foucault C, Brouqui P, Raoult D. *Bartonella quintana* characteristics and clinical management. Emerg Infect Dis. 2006;12:217–23. 10.3201/eid1202.050874 16494745 PMC3373112

[5] Haller JS Jr. Trench foot—a study in military-medical responsiveness in the Great War, 1914–1918. West J Med. 1990;152:729–33.https://www.ncbi.nlm.nih.gov/entrez/query.fcgi?cmd=Retrieve&db=PubMed&list_uids=1972307&dopt=Abstract 1972307 PMC1002454

[6] Lang N, Soskolne WA, Greenstein G, Cochran D, Corbet E, Meng HX, et al. Consensus report: necrotizing periodontal diseases. Ann Periodontol. 1999;4:78. 10.1902/annals.1999.4.1.78

[7] Tschanz DW. Typhus fever and the destruction of the Grand Army. Command. 1991;11:22–5.

[8] Wever PC, van Bergen L. Death from 1918 pandemic influenza during the First World War: a perspective from personal and anecdotal evidence. Influenza Other Respi Viruses. 2014;8:538–46. 10.1111/irv.12267 24975798 PMC4181817

